# Pathological and Genomic Findings of *Erysipelothrix rhusiopathiae* Isolated From a Free-Ranging Rough-Toothed Dolphin *Steno bredanensis* (Cetacea: Delphinidae) Stranded in Korea

**DOI:** 10.3389/fvets.2022.774836

**Published:** 2022-05-06

**Authors:** Kyunglee Lee, Seon Young Park, Hwi Won Seo, Yuna Cho, Seok-Gwan Choi, Seunghyun Seo, Wonmin Han, Nam-Kyung Lee, Hyemin Kwon, Jee Eun Han, Ji Hyung Kim

**Affiliations:** ^1^Cetacean Research Institute, National Institute of Fisheries Science, Ulsan, South Korea; ^2^Infectious Diseases Research Center, Korea Research Institute of Bioscience and Biotechnology, Daejeon, South Korea; ^3^Aquaplanet Co. Ltd., Seoul, South Korea; ^4^Biotherapeutics Translational Research Center, Korea Research Institute of Bioscience and Biotechnology, Daejeon, South Korea; ^5^College of Veterinary Medicine, Kyungpook National University, Daegu, South Korea; ^6^Department of Food Science and Biotechnology, Gachon University, Seongnam, South Korea

**Keywords:** Clade 1, free-ranging cetaceans, genome, serovar 2/15, zoonotic pathogens

## Abstract

Erysipelas, caused by *Erysipelothrix rhusiopathiae*, is considered one of the most serious infectious diseases of captive and free-ranging cetaceans worldwide, as these animals are known to be highly susceptible to the bacterial infections. The potential diversity between *E. rhusiopathiae* isolates from captive cetaceans has been previously described; however, the microbiological features of the free-ranging cetacean isolates remain unclear. Here, we describe a case of bacteremia in a rough-toothed dolphin (*Steno bredanensis*) caused by *E. rhusiopathiae*. Additionally, we present the first genomic features of the bacteria from free-ranging cetacean individuals. Histopathological and microbial examinations revealed that *E. rhusiopathiae* caused bacteremia and systemic infection in the dolphin. The genome of the isolated *E. rhusiopathiae* strain KC-Sb-R1, which was classified as Clade 1 possessing *SpaB* gene, was clearly differentiated from the other swine-isolated *E. rhusiopathiae*, and the comparison of its serovar-defining chromosomal region revealed that our isolate was greatly similar to those of other previously reported serovar 2/15 isolates, including the captive-dolphin isolate. Moreover, most of the potential virulence factors in the strain KC-Sb-R1 were similar to those in the strain Fujisawa. Further, a potential cytotoxicity of the isolate was confirmed, suggesting that marine mammal-isolated *E. rhusiopathiae* could possess strong pathogenic potential in other animals, including humans. These results would further increase our understanding on the risk factors for controlling zoonotic pathogens of emerging infectious diseases in captive or free-ranging cetaceans, and also provide important insight into the diversity of *E. rhusiopathiae* in animals.

## Introduction

The genus *Erysipelothrix* is a ubiquitous Gram-positive bacillus with worldwide distribution, and recent phylogenomic studies have described the presence of five known species: *Erysipelothrix rhusiopathiae, E. tonsillarum, E. inopinata, E. larvae*, and *E. piscisicarius* (1). Among these, *E. rhusiopathiae*, which is the most well-known and industrially important member of the genus, is the causative agent of erysipelas in a wide variety of domesticated and wild animal species, including birds, mammals, reptiles, fish, and arthropods (2, 3). Moreover, the species are known to be associated with zoonotic infections in humans, causing a skin disease known as “erysipeloid,” which can lead to septicemia and endocarditis (4, 5). The potential diversity of *E. rhusiopathiae* has been previously verified mainly based on the serotype, surface protective antigen (Spa) type, and pulsed-field gel electrophoresis-based genotype (6–8). A recent comparative genomic analysis *of E. rhusiopathiae* from a wide range of hosts revealed the presence of four genomic clades (i.e., clades 1, 2, 3, and intermediate), suggesting that the species are more complex than previously assumed (9).

*Erysipelothrix rhusiopathiae* infection is considered one of the most serious infectious diseases of cetaceans, as the animals are known to be highly susceptible to *E. rhusiopathiae* infections; from the first report in porpoise (10), its infection has been recognized in up to 10 different captive or free-ranging (or wild) cetacean species (11). Clinical symptoms of *E. rhusiopathiae* infection in cetaceans have been reported in the classic dermatologic (12) and acute septicemic forms (13–17); however, the disease course is predominantly acute and commonly fatal (18). Although several experimental vaccination trials against *E. rhusiopathiae* in captive cetaceans have yielded encouraging results (19, 20), the potential diversity between the isolates from captive cetaceans has been previously addressed using spa-typing and comparative genomics (9, 21). However, the microbiological and genomic features of *E. rhusiopathiae* isolates causing fatal infection in free-ranging cetaceans remain unclear owing to the limitations of post-mortem analyses of stranded individuals and the lack of reports describing its isolation.

Since 2016, we have investigated the emergence of microbial pathogens infecting cetacean species present in the coastal waters of the Republic of Korea. Our aim was to identify potential pathogens that can colonize and establish infection in marine mammals for conservation. The present study describes a case with pathological and microbiological features of *E. rhusiopathiae*, which caused bacteremia and systemic infection in a stranded free-ranging, rough-toothed dolphin (*Steno bredanensis*). To the best of our knowledge, this is the first case of *E. rhusiopathiae* infection in *S. bredanensis*, and is also the first report describing the genomic features of a free-ranging cetacean-originated isolate causing fatal infection.

## Materials and Methods

### Case Description

The stranded dolphin in this study was cared for (or managed) in a captive environment and handled according to Korean law (Act on the Management of Zoos and Aquariums, Act 14227/2016). All the samples obtained (*in vivo* diagnostic swabs and blood, and post-mortem samples) were collected according to the above and within the Korean law (Wildlife Protection and Management Act, Act 13882/2016), which establishes the management objectives and prescriptions to maintain the species under human care. The study was reviewed and approved by the Ethics and Welfare Committee (Approval Number: 2017-Animal Experiment-15) of the National Institute of Fisheries Science, Ministry of Oceans and Fisheries, Korea.

In April 2018, a rough-toothed dolphin (*S. bredanensis*) was stranded in a wharf of Yeosu, Korea. The dolphin was lethargic and unable to swim back to the sea, and was subsequently rescued and transferred to the local marine animal rescue center (Hanwha Aquaplanet, Yeosu, Korea). The scratches on its back and *Xenobalanus globicipitis*, observed on the edge of the fin, were treated, and it was subsequently hospitalized in a treating pool. The animal presented signs of lethargy, tachypnea (>12–15/min), with vomiting of undigested fish and nematode parasites. Blood sampling was performed on the tail fin, and enrofloxacin and phenylbutazone were intramuscularly administered. Moreover, topical ointment was applied on the wounds of the back and trunk. The next morning, the dolphin was dead, and the body was stored in ice at room temperature until the post-mortem.

### Necropsy and Histopathology

Full necropsy and measurement of the whole body were performed 1 day following the death. The internal organs were examined representative samples from multiple organs (Voucher No. CRI008296 deposited in the Cetacean Research Institute, National Institute of Fisheries Science, Korea) were fixed in 10% neutral formalin and subjected to histological examination. Slides were prepared using conventional histological techniques, and sections were cut to 5-μm thickness and stained with hematoxylin and eosin.

To confirm the potential infection of *Brucella* spp. and morbillivirus in dolphins, polymerase chain reaction (PCR) was performed on frozen samples (i.e., liver, lungs, lymph nodes, kidney, pancreas, and spleen) using two previously published protocols (22, 23).

### Bacterial Isolation and Identification

Sterile swabs were collected from the blowhole, urogenital slit, anus, blood, kidney, and pleural fluid of the carcass during necropsy. The bacteria were isolated using the standard dilution plating technique using brain heart infusion (BHI) agar (Difco, Detroit, MI, USA) and sheep blood agar (Synergy Innovation, Seongnam, Korea), followed by incubation at 37°C for 24 h. Special cultures for screening *Vibrio* spp., *Salmonella* spp., *Listeria* spp., and *Brucella* spp. were also performed as previously described (24).

During bacterial culture, small whitish opaque colonies were isolated from the examined samples, and subcultured three times. The isolated bacteria were identified using 16S rRNA sequencing analysis. Bacterial genomic DNA was isolated using the DNeasy Blood & Tissue Kit (Qiagen Korea Ltd., Seoul, Korea) according to the manufacturer's instructions. The 16S rRNA gene was amplified using universal primers 27F and 1492R, and the amplicons were sequenced using universal primers 785F and 907R. Overall, the PCR amplification of the bacterial 16S rRNA gene was performed using the Maxime PCR PreMix kit (Intron Biotechnology Ltd., Seongnam, Korea), and all PCR products were purified using the QIAquick Gel Extraction Kit (Qiagen Korea Ltd.) before sequencing by Macrogen Inc. (Seoul, Korea). The PCR profile consisted of 3 min at 94°C, followed by 35 cycles of 60 s at 94°C, 60 s at 55°C, 3 min at 72°C, and a final extension for 3 min at 72°C. All the obtained isolates were stored in BHI broths (Difco) supplemented with 10% glycerol at −80°C until further use.

### Detection of *Erysipelothrix* Using a PCR Assay

PCR examination was performed to detect the presence of bacteria belonging to the genus *Erysipelothrix* in the collected organs (testicle, kidney, and rectum) and body fluids (blood and pleural fluid). Total genomic DNA of the samples was isolated using the DNeasy Blood and Tissue Kit (Qiagen Korea Ltd.) according to the manufacturer's instructions. Multiplex PCR assays were conducted using the primer sets ERY-1F/ERY-2R (2,210 bp, targeting specific regions for *E. rhusiopathiae* chromosomal DNA) and MO101/ERS-1R (719 bp, targeting the highly conserved region of 16S rRNA of genus *Erysipelothrix*) based on the report by Yamazaki (25), and the amplicons were sequenced at Macrogen Inc. (Seoul, Korea) and compared with other *Erysipelothrix* strains in the GenBank database by BLAST searches (www.ncbi.nlm.nih.gov/BLAST).

### Antimicrobial Susceptibility Test

Antimicrobial susceptibility of the isolated bacteria was evaluated according to the testing guidelines and interpretive breakpoints for *E. rhusiopathiae* in the M45 document from the Clinical and Laboratory Standards Institute (CLSI) (26). The minimum inhibitory concentrations (MICs) were determined using MIC Evaluator Strips (Liofilchem®, Teramo, Italy) of 11 antimicrobial agents from six classes as follows: penicillin (ampicillin, 256–0.016 μg; penicillin, 256–0.016 μg), cephalosporins (cefepime, 256–0.016 μg; cefotaxime, 256–0.016 μg; ceftriaxone, 256–0.016 μg), carbapenems (imipenem, 256–0.016 μg; meropenem, 256–0.016 μg), macrolides (erythromycin, 256–0.016 μg), fluoroquinolones (ciprofloxacin, 256–0.016 μg; levofloxacin, 256–0.016 μg), and lincosamides (clindamycin, 256–0.016 μg). The MIC tests were conducted on the Muller-Hilton blood agar (Synergy Innovation) at 37°C for 24 h, and *Streptococcus pneumoniae* ATCC 49619 was used for quality control.

### Genome Sequencing and Analyses

To confirm the exact species and types of the isolates in the genus *Erysipelothrix*, the bacterial genome was sequenced using a hybrid approach on a PacBio RS II system (Pacific Biosciences, Menlo Park, CA, USA) by constructing a 20-kb SMRTbell™ template library, and the HiSeq 2000 system (Illumina, San Diego, CA, USA) by preparing a DNA library using the TruSeq Nano DNA Library Prep Kit (Illumina). The genome assembly of the filtered PacBio reads (872,887,747 bp; 77,271 reads) was performed using the HGAP (v3.0) pipeline, and the Illumina paired-end 150-bp reads (1,810,892,868 bp, 11,992,668 reads) were mapped using BWAMEM (v0.7.15), while the errors were corrected by Pilon (v1.21) using default parameters. Annotation was performed using the NCBI Prokaryotic Genome Annotation Pipeline (http://www.ncbi.nlm.nih.gov/books/NBK174280/). To determine the exact species of the *Erysipelothrix* isolate, the *rpoB* gene, which encodes the β-subunit of DNA-dependent RNA polymerase, was manually searched from the genome and was aligned with the representative sequences from the strain types of five *Erysipelothrix* species in the GenBank database using ClustalX (v2.1) (27) and BioEdit Sequence Alignment Editor (v7.1.0.3; Bioedit, Manchester, UK) (28). Then, the datasets were phylogenetically analyzed using MEGAX (v10.0) (29). The phylogenetic trees were constructed using the maximum-likelihood (ML) method, and the reliability of the trees was assessed using 1,000 bootstrap replicates. To assess the genomic relatedness of the isolate with other *Erysipelothrix* species, the average nucleotide identity (ANI) was analyzed using OrthoANI (https://www.ezbiocloud.net/tools/orthoani) against several other strain types of the genus available in the GenBank database. To determine the Spa type of the isolate, putative *spa* genes were manually searched in the sequenced genome using BLAST (www.ncbi.nlm.nih.gov/BLAST), and compared with the antigenically different Spa proteins (SpaA, B, and C) of the species. Moreover, to determine the potential serotype of the isolate, the serovar-defining chromosomal region of the strain KC-Sb-R1 was manually searched against those previously-identified regions from the strain Fujisawa (serovar 1a, ERH_1438 to ERH_1451) (30) using BLAST, and further compared with the 28 serovar strains of *Erysipelothrix* species (31).

Putative virulence-associated genes were preliminarily screened by searching against the virulence factor (http://www.mgc.ac.cn/VFs/), and were ultimately identified on manual comparison with the *E. rhusiopathiae* strain Fujisawa (NC_015601.1). The presence of genetic determinants related to antibiotic resistance was screened using the Comprehensive Antibiotic Resistance Database (https://card.mcmaster.ca/) and the ARG-ANNOT database (http://en.mediterranee-infection.com/article.php?laref=283&titre=arg-annot). PHASTER (http://phaster.ca/) analysis was performed to detect the prophages in the genome.

### Evaluation of the Potential Bacterial Cytotoxicity

The Quanti-Max WST-8 Cell Viability Assay Kit (Biomax Ltd., Heidelberg, Germany) was used to analyze cell viability according to the manufacturer's instructions with some modifications. The mouse macrophage cell line Raw 264.7 (ATCC TIB-71) and the human airway epithelial cell line Calu-3 (ATCC HTB-55) were maintained at 37°C in Dulbecco's Modified Eagle's Medium (Corning Corp., New York, NY, USA) supplemented with 10% heat-inactivated fetal bovine serum (Corning Corp.) and 1x Antibiotic-Antimycotic (Gibco, Waltham, MA, USA) in an atmosphere of 5% CO_2_ saturated with water. In the case of Raw 264.7 and 2 mM Calu-3, Glutagro (L-glutamine; Corning Corp.), 1 mM sodium pyruvate (Gibco), and 0.1 mM MEM NEAA (non-essential amino acids; Gibco) were additionally provided in the culture media. Raw 264.7 (3 × 10^3^/well) and Calu-3 (5 × 10^3^/well) were seeded into 96-well plates in Dulbecco's Modified Eagle's Medium with 10-mM HEPES buffer (Gibco). Cells in each well were suspended in 10-μL fresh media containing various concentrations (10^2^, 10^4^, 10^5^, 10^6^, and 10^8^ CFU/mL) of the strain KC-Sb-R1 and incubated at 37°C for 24 and 48 h, respectively. After incubation, 5 μL WST-8 reagent was added to each well, and the samples were incubated for 30 min at 37°C. The cell viability of each plate was measured at 450 nm using a microplate reader (SpectraMax M5e, Molecular Devices, San Jose, CA, USA). All assays were performed in triplicate.

### Nucleotide Sequences Deposition

The nucleotide sequences of the 16S rRNA from the *E. rhusiopathiae* strain KC-Sb-R1 were deposited in the GenBank database under the accession number MH718800.1. The complete genome sequence of the strain KC-Sb-R1 has been deposited in GenBank under the accession number NZ_CP033601.1.

## Results

### Necropsy and Histopathology

**Figure 1:**
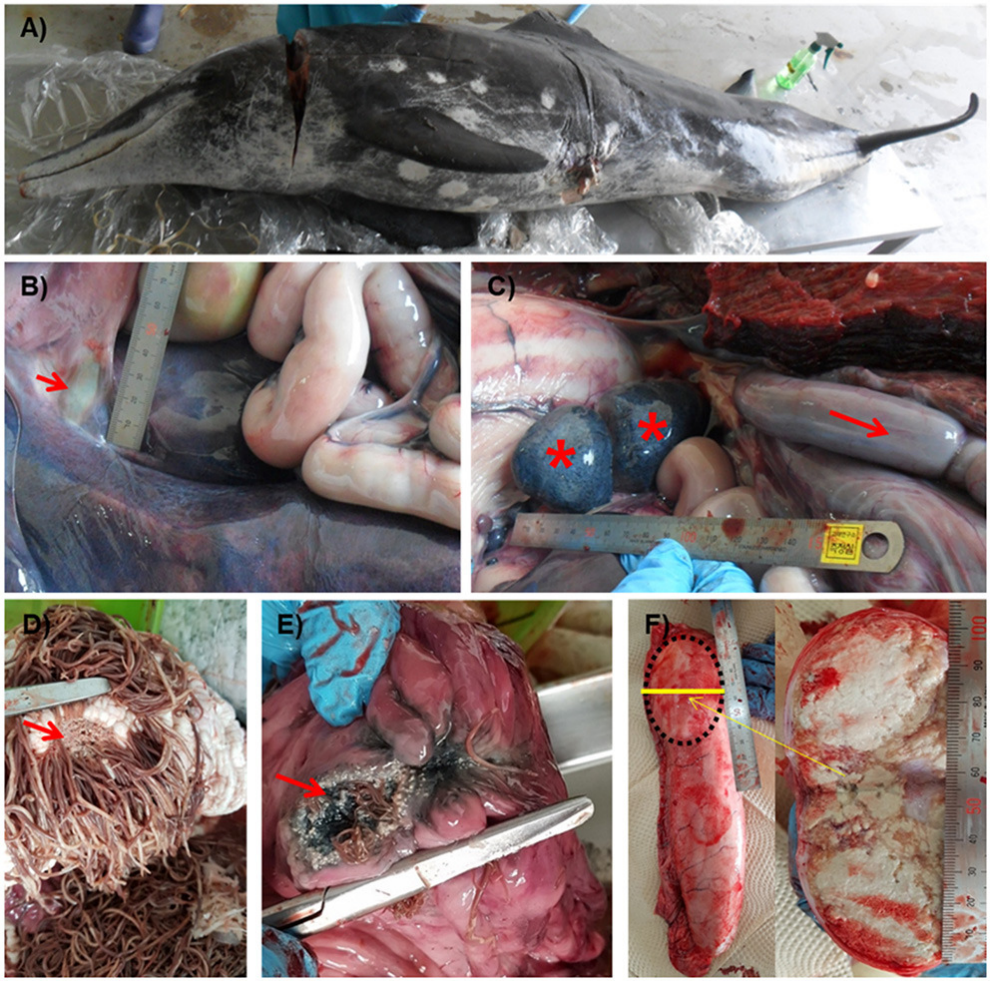
Gross lesions of a *Steno bredanensis* that died from septicemia caused by *Erysipelothrix rhusiopathiae*. **(A)** Overall external features of the dead dolphin. **(B)** The bile duct to the duodenum. Trematodes were observed in the duct, liver, and pyloric stomach (arrow). **(C)** Spleen and accessory spleen with petechiae (asterisk), enlarged and congested intestine (arrow). **(D,E)** Severe *Anisakis* infection formed ulcers (arrow) on the forestomach **(D)** and main stomach **(E)** walls. **(F)** Caseous exudate fills capsule formed in the testis.

The stranded rough-toothed dolphin was a male dolphin weighted 122.6 kg with a size of 223.5 cm. It was assumed to be an aged adult dolphin with white patterns around the mouth and worn teeth. Blood analysis results revealed moderate dehydration, anemia, and chronic inflammation. Scratches on the back, a cut with hypertrophied margin on the left abdominal trunk, and several healed cookie-cutter shark marks were observed (Figure 1A). Major gross findings in the dolphin included marked edema and congestion of the intestine, generalized lymphadenomegaly, and foam in the trachea with lung congestion. Some trematodes were found in the bile duct (Figure 1B). The enlarged intestine filled with gas was positioned cranially in the abdominal cavity (Figure 1C). The fore and main stomachs were filled with clumps of *Anisakis* spp. (>500 g), with ulcer formation (Figures 1D,E). The rectum was filled with dark bloody feces. Caudal one-third of the right testis was hardened and filled with caseous exudate (Figure 1F). No rhomboid-shaped skin lesions of typical erysipelas in cetaceans (11) were observed on the skin of the examined dolphin.

**Figure 2:**
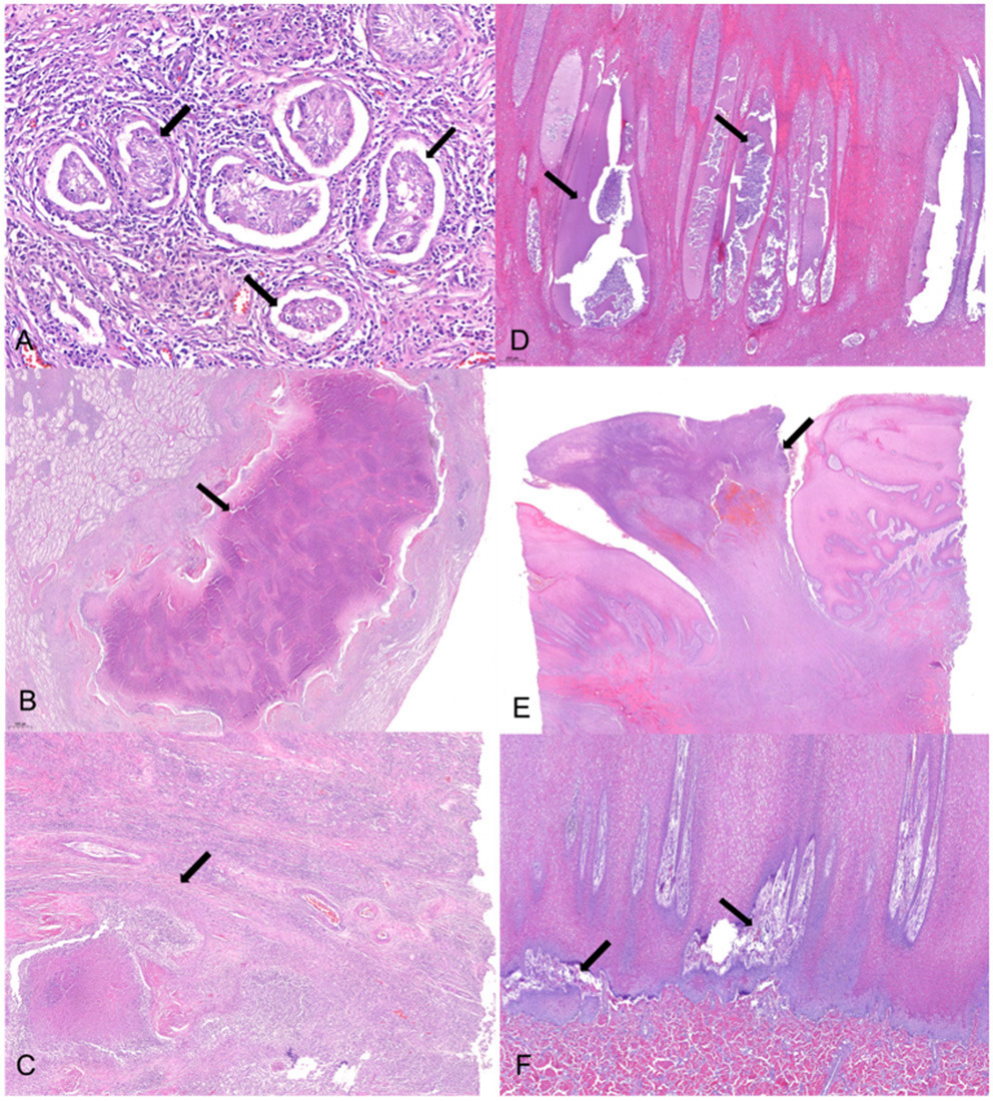
Histopathological changes of the testicular and skin tissues. **(A)** Tubular degeneration with partial or complete loss (arrow) of germ cell layers were observed diffusely in the testis tissue. **(B)** Abscess (arrow) with polymorphonuclear inflammatory cell and necrotic debris enclosed with fibrotic tissue was observed in a broad portion of the testicular lobules. **(C)** Fibrosis (arrow) of the interstitial, perivascular, and peritubular regions was concurrently observed with necrosis and inflammation, resulting in tubular atrophy. **(D)** Pustules (arrow) containing neutrophils and cell debris were diffusely distributed in the thickening stratum spinosum epidermal layer. **(E)** Severe chronic focal ulcerative dermatitis (arrow) with hemorrhage was observed in the epidermal layer expanding into the dermis layer. **(F)** Papillary layer of dermal-epidermal junction was irregularly arranged (arrow) in the damaged skin. Hematoxylin and eosin stain; 4 × **(E)**, 20 × **(B,D,F)**, 40 × **(C)**, and 100 × **(A)** magnification.

Histopathological lesions were observed both in the testes and skin (Figure 2). Tubular degeneration with partial or complete loss of germ cell layers was diffusely observed in the testis tissue (Figure 2A). Moreover, diffuse infiltration of mixed inflammatory cells was observed from the capsular connective tissue to the seminiferous tubules, which was associated to abscess containing polymorphonuclear inflammatory cells and necrotic debris (Figure 2B). In addition, tubular atrophy with interstitial fibrosis was observed (Figure 2C). In the case of the skin, pustules containing neutrophils and cell debris were diffusely distributed in the thickened stratum spinosum epidermal layer (Figure 2D). Severe chronic focal ulcerative dermatitis with hemorrhage was also observed in the epidermal layer, expanding into the dermis layer (Figure 2E). Moreover, the papillary layer of the dermal-epidermal junction was irregularly arranged in the damaged skin (Figure 2F).

### *E. rhusiopathiae* Caused Systemic Infection in *S. bredanensis*

During the bacterial isolation process, small whitish opaque α-hemolytic colonies were simultaneously isolated from the urogenital slit, anus, blood, kidney, and pleural fluids. Based on the 16S rRNA sequencing analysis, the isolates showed 99.9–100% sequence identity within themselves and were very similar (>99%) to those of the other *Erysipelothrix* strains available in the GenBank database. This finding suggested that the dolphin-isolated strain in this study could also be identified as members of the genus *Erysipelothrix*. Moreover, there was no evidence of growth of *Vibrio* spp., *Salmonella* spp., *Listeria* spp., or *Brucella* spp. in selective or enriched media from internal organs and body fluids, and the PCR finding was negative for *Brucella* spp. and morbillivirus. *Erysipelothrix* was the only species isolated in large numbers by routine culture of the sampled organs in the dolphin.

A multiplex PCR assay using the primer sets MO101/ERS-1R and ERY-1F/ERY-2R successfully amplified the nucleotide fragments of 2,210 bp and 719 bp from all the *Erysipelothrix* isolates in this study, thus, indicating that the isolates could be classified as *E. rhusiopathiae*. Moreover, as expected, the two positive amplicons were also found in all the collected organ and body fluid samples, suggesting that the bacteria might have caused systemic infection in the dolphin (data not shown). The representative *E. rhusiopathiae* strain (designated KC-Sb-R1) isolated from the blood of the dolphin was used for further examination in this study.

The resistance profiles of the *E. rhusiopathiae* strain KC-Sb-R1 to several antimicrobial classes were evaluated using the MIC methods based on the CLSI guidelines. In this study, the *E. rhusiopathiae* isolates were not resistant to any of the tested antibiotics; the overall antimicrobial resistance profiles of the strain KC-Sb-R1 are summarized in Supplementary Table S1.

### Genome of *E. rhusiopathiae* Strain KC-Sb-R1 Revealed Unique Features

**Figure 3:**
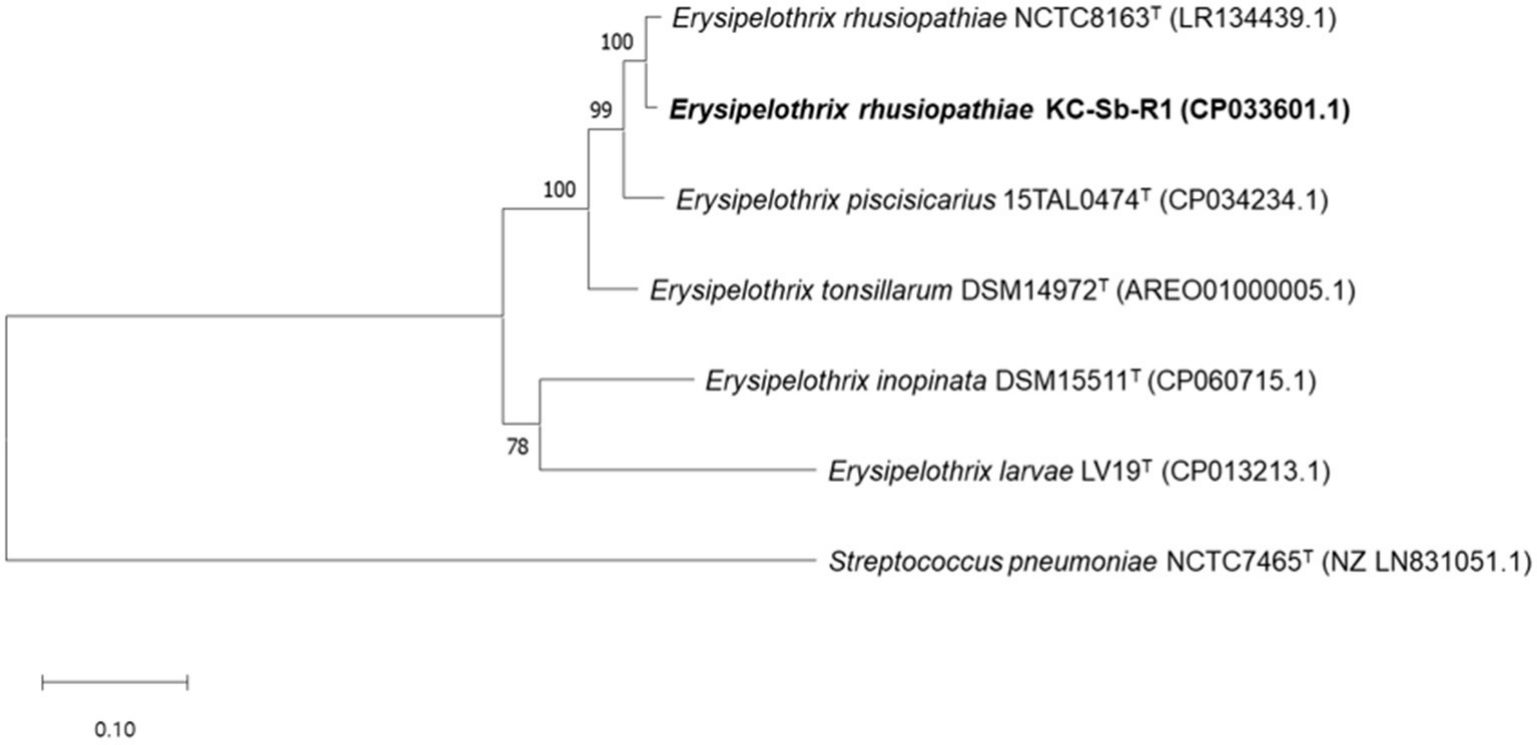
Maximum-likelihood tree based on the nucleotide sequences of *rpoB* encoding the β-subunit of DNA-dependent RNA polymerase in *Erysipelothrix rhusiopathiae* strain KC-Sb-R1 to five other strain types of the *Erysipelothrix* species and the outgroup *Streptococcus pneumoniae* NCTC7465^T^. The scale bar represents 0.1 nucleotide substitutions per site.

The fully assembled complete genome of the strain KC-Sb-R1 was 1,771,674 bp long (G+C content, 36.6%); plasmids were not detected. The annotated genome possessed 1,713 genes, 1,633 coding sequences, 21 rRNAs (5S, 16S, and 23S), 55 tRNAs, and four non-coding RNAs. Using the obtained genome, we first verified the potential usability of the *rpoB* gene for single gene-based species identification. The *rpoB* gene (3,777 bp, locus_tag: EEY85_08105) of KC-Sb-R1 was manually searched and used for further phylogenetic analysis. The resultant phylogeny based on the *rpoB* gene of the isolate showed a clear distinction from the other four *Erysipelothrix* species (i.e., *E. tonsillarum, E. piscisicarius, E. inopinata*, and *E. larvae*) (Figure 3).

Then, we conducted the sequence-based characterization of the predicted *SpaB* gene in the strain KC-Sb-R1. The *SpaB* gene (1,893 bp, locus_tag: EEY85_08350) of the strain KC-Sb-R1 was almost identical (>99.9%) to that of the strain Sapporo, which was isolated from human erysipelas infection in Japan (32). Additionally, the deduced amino acid sequence of the *SpaB* gene was very similar (96.4–99.8%) to that of other *SpaB* genes of *E. rhusiopathiae* isolates available in the GenBank database, and clearly differentiated from *SpaA* and *SpaC*.

Subsequently, to assess the overall genome similarity between the strain KC-Sb-R1 and other *E. rhusiopathiae* genomes, the OrthoANI algorithm was adapted in this study. Similar to the previous report of Grazziotin et al. (1), the strain KC-Sb-R1 showed >96% ANI values against all the other *E. rhusiopathiae* genomes available in the GenBank database, including the newly released *E. rhusiopathiae* genomes (the ZJ, SE38, and G4T10 strains), thus, indicating that our isolate could also be confirmed as a member of *E. rhusiopathiae*. However, the strain KC-Sb-R1 was clearly differentiated from the swine-isolated *E. rhusiopathiae* by showing >3% ANI value differences (Supplementary Table S2).

**Figure 4:**
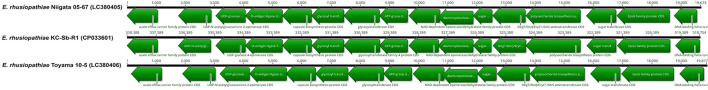
Comparison of the serovar-defining chromosomal regions of the *E. rhusiopathiae* strain KC-Sb-R1 and other serovar 2/15 strains (Niigata 05-67 and Toyama 10-5)* available in the GenBank database. *The strain Quitz 262 was not included, as its complete nucleotide sequence is not available in the GenBank database.

Then, to determine the potential serotype of the isolate, the genes homologous to ERH_1438 and ERH_1451 were preliminarily searched and, respectively, found at the locus_tags EEY85_01660 (337345..338388) and EEY85_01585 (318754..319575) of the isolate; in total, 19,634 bp sequences of the serovar-defining chromosomal region were finally obtained between the predicted genes. The sequence analysis revealed that the gene content and organization of the chromosomal regions of the strain KC-Sb-R1 was very similar to those of other previously reported serovar 2/15 isolates, Toyama 10-5 (>99.9%, wild boar origin) and Niigata 05-67 (96.4%, pig origin), which were isolated in Japan (33) (Figure 4). Although one case of fatal infection caused by serovar 2/15 *E. rhusiopathiae* (strain Quitz 262) has been reported in captive dolphin in the USA (9, 21, 34), we could not directly compare the serovar-defining chromosomal region between the strains KC-Sb-R1 and Quitz 262, as its complete nucleotide sequence is not available in the GenBank database. Therefore, the genomic relatedness of the strains KC-Sb-R1 and Quitz 262 was further analyzed using direct comparison of eight housekeeping genes [galactokinase (*galK*), glycerol-3-phosphate-dehydrogenase (*gpsA*), d-lactate dehydrogyenase (*ldhA*), ribose-phosphate-pyrophosphokinase (*prsA*), phosphate acetyl-transferase (*pta*), adenylosuccinate synthetase (*purA*), recombinase A (*recA*), and DNA gyrase B (*gyrB*)] (34); the results revealed that the two *E. rhusiopathiae* stains are closely related: 100% identities in *gpsA, gyrB*, and *purA*; 99.9% identities in *recA*; 99.7% identities in *galK*; 99.6% identities in *prsA*; 98.9% identities in *pta*; and 95.0% identities in *ldhA*.

Finally, the presence of potential virulence-associated genes was identified by manual comparison with strain Fujisawa (NC_015601.1). Overall, most of the potential virulence factors in KC-Sb-R1 were similar to those in the strain Fujisawa, including surface and antioxidant proteins, phospholipases, and hemolysins; however, several genes related to lysophospholipase, neuraminidase, and biofilm formation were not found in the genome (Table 1). Moreover, the strain KC-Sb-R1 did not possess plasminogen- (ERH_0768) and collagen-binding proteins (ERH_1436); however, the two recently-identified virulence-associated surface proteins (ERH_0728 and ERH_1472), which are involved in bacterial adherence to porcine endothelial cells (35), were detected in the genome. Additionally, no putative antimicrobial resistance-related genes were detected in the genome.

**Table 1:** Comparisons of potential virulence factors of the *E. rhusiopathiae* strains Fujisawa and KC-Sb-R1.

***E. rhusiopathiae*** **strain Fujisawa**		***E. rhusiopathiae*** **strain KC-Sb-R1**	
**Locus tag**	**Predicted function (gene)**	**Locus tag**	**Identity (%)**
**Surface proteins**			
ERH_0075	Collagen-binding protein	EEY85_RS08445	93.3
ERH_0094	Protective antigen (*spaA.1*)	ND*	-
ERH_0150	Hyaluronidase (*hylA*)	EEY85_RS08080	97.7
ERH_0161	Peptidase M14	EEY85_RS08020	97
ERH_0201	Pectin lyase fold-containing protein	EEY85_RS07735	92.9
ERH_0221	Glycoside hydrolase, family 16	EEY85_RS07635	96.8
ERH_0260	Proteinase	EEY85_RS07470	93.3
ERH_0278	Unknown	EEY85_RS07400	94.7
ERH_0299	Neuraminidase (*nanH.1*)	EEY85_RS07290	93.7
ERH_0407	Choline-binding protein (*cbpA*)	EEY85_RS06730	92.9
ERH_0561	Glycosyl hydrolase, family 85	EEY85_RS05920	95.6
ERH_0668	Biofilm formation, protective antigen (*rspA*)	EEY85_RS05620	90.7
ERH_0669	Biofilm formation (*rspB*)	EEY85_RS05615	95.3
ERH_0728	Internalin-like	EEY85_RS05350	96.5
ERH_0765	Hyaluronidase (*hylB*)	EEY85_RS05150	90.6
ERH_0768	Adhesin, Plasminogen-binding protein (*cbpB*)	ND*	-
ERH_0777	Dipeptidase	EEY85_RS05090	92.8
ERH_1139	5 –Nucleotidase *(ushA)*	EEY85_RS03210	95.4
ERH_1210	Hyaluronidase *(hylC)*	EEY85_RS02810	98.8
ERH_1258	Unknown	ND*	-
ERH_1436	Collagen-binding protein	ND*	-
ERH_1454	Unknown	EEY85_RS01575	80.3
ERH_1472	Internalin-like	EEY85_RS01510	96.7
ERH_1687	Biofilm formation (*rspC*)	EEY85_RS00385	95.2
**Antioxidant proteins**			
ERH_0162	Thiol peroxidase (*tpx*)	EEY85_RS08015	97.5
ERH_0175	Alkyl-hydroperoxide reductase (*ahpC*)	EEY85_RS07915	97.1
ERH_0356	Glutaredoxin (*nrdH*)	EEY85_RS06985	100
ERH_0375	Thioredoxin (*trxA.1*)	EEY85_RS06895	99.7
ERH_1065	Superoxide dismutase (*sodA*)	EEY85_RS03630	98
ERH_1311	Thioredoxin-disulfide reductase (*trxB.1*)	EEY85_RS02350	97.5
ERH_1345	Alkylhydroperoxide reductase (*ahpD*)	EEY85_RS02175	98.6
ERH_1500	Thioredoxin (*trxA.2*)	EEY85_RS01325	99.7
ERH_1541	Thioredoxin-disulfide reductase (*trxB.2*)	EEY85_RS01105	97.2
**Phospholipase**			
ERH_0072	Patatin-like phospholipase	EEY85_RS08505	96.7
ERH_0083	Phospholipase/ Carboxylesterase family	EEY85_RS08405	92.8
ERH_0148	Lysophospholipase (*pldB*)	EEY85_RS08090	99
ERH_0333	Cardiolipin synthetase (*cls*)	EEY85_RS07130	96.7
ERH_0334	Patatin-like phospholipase	EEY85_RS07125	96.5
ERH_0347	Phospholipase/ carboxylesterase	EEY85_RS07030	99.5
ERH_0388	Phospholipase D	EEY85_RS06835	94.9
ERH_1214	Lysophospholipase	EEY85_RS02790	99.1
ERH_1433	Lysophospholipase	ND*	-
**Hemolysins**			
ERH_0467	Hemolysin-related protein	EEY85_RS06410	97.7
ERH_0649	Hemolysin III	EEY85_RS05720	91.4
**Other extracellular proteins/enzymes**			
ERH_0761	Neuraminidase (*nanH.2*)	ND*	-
ERH_1034	Fibronectin-binding protein	EEY85_RS03835	94.3
ERH_1356	Adhesin	EEY85_RS02120	97.2
ERH_1467	Biofilm formation	ND*	-

*ND, Not detected.

During the search for a putative prophage sequence using PHASTER, only one unique phage sequence (14.9kb, 502467..517436), which showed no pairwise identity with other available *E. rhusiopathiae* strains in the GenBank database, was identified in the genome of the strain KC-Sb-R1 (Supplementary Table S3).

### Potential Cytotoxicity of *E. rhusiopathiae* Strain KC-Sb-R1

To evaluate the virulence potential of *E. rhusiopathiae* strain KC-Sb-R1, we investigated the cytotoxic activity using human- and murine-derived cell lines by measuring cell viability against the bacterial exposure. The potential viability of Raw 264.7 and Calu-3 cells indicated significant differences, based on the treatment with different concentrations of *E. rhusiopathiae* strain KC-Sb-R1, compared with the control group (Supplementary Figure S1). Treatment of the two cell lines with the strain KC-Sb-R1 indicated significant reductions in their viability by presenting dose-dependent increases in potential cytotoxicity; >30% loss of murine cell viability was observed within 24 h post-bacterial exposure (*p* <0.05) and significant reductions of viabilities (>20%, *p* < 0.001) were observed after 48 h of incubation inoculated with 10^8^ CFU/mL of the strain KC-Sb-R1.

## Discussion

To date, several bacterial species have been recognized as causative agents of emerging infectious diseases in marine mammals; *E. rhusiopathiae* is now considered as one of the most important zoonotic pathogen causing fatal infections in captive and free-ranging cetaceans worldwide rather in pinnipeds (36). To date, a number of cetacean species have been reported to be susceptible to *E. rhusiopathiae* infections: bottlenose dolphin (*Tursiops truncatus*), spotted dolphin (*Stenella plagiodon*), long-finned pilot whale (*Globicephala melas*), Indo-Pacific bottlenose dolphin (*T. aduncus*), white-beaked dolphin (*Lagenorhynchus albirostris*), Pacific white-sided dolphin (*L. obliquidens*), beluga whale (*Delphinapterus leucas*), Risso's dolphin (*Grampus griseus*), killer whale (*Orcinus orca*), harbor porpoise (*Phocoena phocoena*), and southern right whale (*Eubalaena australis*) (11, 17). To our knowledge, this is the first report of *E. rhusiopathiae* infection in the rough-toothed dolphin (*S. bredanensis*), as the exact species of the stranded dolphin individual in this study (Voucher No. CRI008296) was previously identified based on the whole-mitochondrial genome sequencing analysis (37). Our findings strongly emphasize that greater awareness is required to reduce the public health risk of un-intended zoonotic infections for humans who come in direct contact with stranded dolphins (e.g., aquarium staffs and veterinarians) during handling and treating of the stranded wild individuals (38).

During the bacterial isolation process, the infection of *E. rhusiopathiae* in the dolphin was confirmed by direct isolation and 16S rRNA sequencing of the bacteria from the urogenital slit, anus, blood, kidney, and pleural fluids. Considering the fact that *E. rhusiopathiae* was isolated from the circulatory system, including blood, and that no other major bacterial and viral pathogens were detected from it, it can be suggested that the bacterial species could have caused bacteremia in the stranded dolphin. Moreover, a multiplex PCR assay (25) using the collected organs (testicle, kidney, and rectum) and body fluids (blood and pleural fluid) was conducted to determine whether *E. rhusiopathiae* caused systemic infection in the dolphin. As expected, the bacteria were detected in all the collected organ and body fluid samples, suggesting that *E. rhusiopathiae* might have caused systemic infection contributing to death of the dolphin without typical clinical signs of erysipelas, as shown in the other cases of free-ranging dolphins (11, 16).

The emergence of *E. rhusiopathiae* can be assumed to be much more plausible in captive than in free-ranging marine mammals owing to their frequent contact with humans. Interestingly, a potential diversity between the bacterial isolates from captive dolphins has been previously described (9, 21). However, compared to the captive animals, only limited cases are currently available owing to the limitations of post-mortem analyses of stranded individuals, and the microbiological and genomic features of *E. rhusiopathiae* isolates in free-ranging animals still remain unclear. Therefore, we have sequenced the genome of the strain KC-Sb-R1 to investigate its relatedness with and uniqueness from other farm animal-originated *E. rhusiopathiae* isolates. Similar to other bacterial genera, the 16S rRNA-based identification system has not fully delineated *Erysipelothrix* species, and multi-locus sequence analyses based on the housekeeping genes (e.g., *galK, gpsA, ldhA, prsA, pta, purA*, and *recA*) can be performed for species delineation (8, 33, 39). Recently, the *rpoB* gene has been suggested as an alternative for the identification of *Erysipelothrix* species, showing clear distinction (1). Our results also revealed that the *rpoB* gene-based identification could be a useful candidate for single gene-based species identification of the genus *Erysipelothrix*.

Originally, the genome sequence of the *E. rhusiopathiae* strain KC-Sb-R1 became publicly available from 2018 in the GenBank database, and certain comparative genomic analyses (i.e., ANI comparison and Spa typing) were recently conducted by Grazziotin et al. (1) during the period this study was conducted and this manuscript was written. Recent comparative genomic analysis of a diverse global collection of *E. rhusiopathiae* isolates from a wide range of host species revealed that the strains can be broadly divided into four clades (Clade 1, 2, 3, and intermediate), and isolates from the cetaceans were classified as Clade 1 (*SpaB* gene-possessing isolates) or Clade 2 (*SpaA* gene-possessing isolates) (9). Based on the results of the analysis by Grazziotin et al. (1), our strain was classified as “Clade 1” with the *SpaB* gene. In this study, we separately conducted the additional ANI value comparison against newly released *E. rhusiopathiae* genomes (the ZJ, SE38, and G4T10 strains). The strain KC-Sb-R1 showed >96% ANI values against all the other *E. rhusiopathiae* genomes available in the GenBank database, thus, indicating that our isolate was within the threshold (ANI values <95–96%) of the current genome-based species delineation system (40–42). However, the strain KC-Sb-R1 was clearly differentiated from the swine-isolated *E. rhusiopathiae*, suggesting that the free-ranging dolphin-originated *E. rhusiopathiae* could be separated from the swine isolates as previously described (9); however, further studies are warranted to fully demonstrate their uniqueness.

Based on the comparisons of the serovar-defining chromosomal region in the strain KC-Sb-R1 against other available *E. rhusiopathiae* isolates, the potential serotype of the isolate was determined to be serovar 2/15. To date, the emergence of pathogenic strains of serovar 2/15 *E. rhusiopathiae* has been rarely reported; however, its fatal infection has also been described in captive dolphins (9, 21, 33). Although we could not directly compare the serovar-defining chromosomal region between the strains KC-Sb-R1 and Quitz 262, the two were confirmed to be closely related, thus, supporting the hypothesis that the cetacean-originated *E. rhusiopathiae* could be separated from the swine isolates; however, more studies are warranted to confirm its potential uniqueness.

Moreover, the potential virulence-associated genes were compared to *E. rhusiopathiae* strain Fujisawa, one of the most thoroughly characterized pathogenic isolate that caused swine erysipelas (43). Overall the potential virulence factors in the strain KC-Sb-R1 were similar to those in the strain Fujisawa. The potential cytotoxic activity of the strain KC-Sb-R1 against human- and mouse-derived cell lines was, respectively, confirmed in this study. These results strongly suggested that the dolphin isolate that caused bacteremia and systemic infection may have strong pathogenic potential in other animals, including humans. Interestingly, the four types of prophage regions (p1, p2, p3, and unique) that have been previously found in the various *E. rhusiopathiae* strains, including several captive cetacean isolates (9), were not detected in the strain KC-Sb-R1, while only one unique phage sequence (14.9 kb) that did not showed identity to the other available *E. rhusiopathiae* strains in the GenBank database was only found in its genome. These results led to the speculation that the pathogenic *E. rhusiopathiae* infecting free-ranging marine mammals may be not so closely related to the other isolates from various animal species, which was also revealed in the ANI comparison results in this study; however, more genomic studies on the isolates from free-ranging marine mammals are warranted to confirm its potential uniqueness in future.

Although several bacterial species, including *E. rhusiopathiae*, have been recognized as causative agents of emerging infectious diseases in cetaceans, the microbiological features of free-ranging individual isolates remain unclear. In this study, we describe the pathological and microbial features of *E. rhusiopathiae* that caused bacteremia and systemic infection in a stranded free-ranging rough-toothed dolphin (*S. bredanensis*). We also present the first genomic features of the isolate from free-ranging cetaceans by providing evidence that the marine mammal-isolated *E. rhusiopathiae* could be differentiated from the other swine-isolated *E. rhusiopathiae*, with strong pathogenic potential causing zoonotic infections, as it was confirmed by the evaluation of potential cytotoxicity in this study. These results would further increase our understanding on the risk factors for controlling zoonotic causative agents of emerging infectious diseases in captive or free-ranging cetaceans, and also provide important insight into the diversity of *E. rhusiopathiae* in animals.

## Data Availability Statement

The datasets presented in this study can be found in online repositories. The names of the repository/repositories and accession number(s) can be found below: https://www.ncbi.nlm.nih.gov/genbank/, MH718800.1; https://www.ncbi.nlm.nih.gov/genbank/, NZ_CP033601.1.

## Ethics Statement

The animal study was reviewed and approved by National Institute of Fisheries Science, Ministry of Oceans and Fisheries, Korea.

## Author Contributions

KL and SP: conceptualization, methodology, conducted the study, data analysis, and writing the manuscript. HS: methodology, conducted the study, and writing the manuscript. YC and S-GC: methodology, conducted the study, and editing the manuscript. SS and WH: supplied animal materials for the study, conducted the study, and editing the manuscript. N-KL: supplied materials for the study and editing the manuscript. HK: conducted the study and editing the manuscript. JH: conceptualization, writing the manuscript, and editing the manuscript. JK: conceptualization, funds, writing the manuscript, supervision, and editing the manuscript. All authors contributed to the article and approved the submitted version.

## Funding

This research was supported by the KRIBB Initiative Programs, the National Research Foundation of Korea (NRF-2020R1I1A2068827) funded by the Ministry of Education and the Collaborative Genome Program of the Korea Institute of Marine Science and Technology Promotion (20180430), and the National Institute of Fisheries Science (R2022033) of the Ministry of Oceans and Fisheries in the Republic of Korea.

## Conflict of Interest

SS and WH were employed by Aquaplanet Co. Ltd. The remaining authors declare that the research was conducted in the absence of any commercial or financial relationships that could be construed as a potential conflict of interest.

## Publisher's Note

All claims expressed in this article are solely those of the authors and do not necessarily represent those of their affiliated organizations, or those of the publisher, the editors and the reviewers. Any product that may be evaluated in this article, or claim that may be made by its manufacturer, is not guaranteed or endorsed by the publisher.
