# Supplementary material for: Pathological and Genomic Findings of Erysipelothrix rhusiopathiae Isolated From a Free-Ranging Rough-Toothed Dolphin Steno bredanensis (Cetacea: Delphinidae) Stranded in Korea
Source: Front Vet Sci. 2022 May 6;9:774836. doi: 10.3389/fvets.2022.774836 (PMC9120913; doi:10.3389/fvets.2022.774836)
Supplement: Supplementary file 1 [file Table_1.docx]

**Supplementary Table 1.**Antibiotic resistance profile of *Erysipelothrix rhusiopathiae* KC-Sb-R1 using the MIC test.

| **Strain** | **Antimicrobial agent [MIC (μg/mL]** | | | | | | | | | | |
| --- | --- | --- | --- | --- | --- | --- | --- | --- | --- | --- | --- |
|  | **Penicillins** | | **Cephems** | | | **Carbapenems** | | **Ma^*^** | **Fluoroquinolones** | | **Lin** |
|  | **Amp** | **P** | **FEP** | **CTX** | **CRO** | **IMI** | **MRP** | **E** | **CIP** | **LEV** | **CD** |
| KC-Sb-R1 | 0.047 | 0.032 | 0.125 | 0.047 | 0.047 | 0.006 | 0.016 | 0.125 | 0.023 | 0.094 | 0.125 |
| CLSI^**^ | ≤0.25 | ≤0.12 | ≤1 | ≤1 | ≤1 | ≤0.5 | ≤0.5 | ≤0.25 | ≤1 | ≤1 | ≤0.25 |

* Ma,Macrolides; Lin,Lincosamides; AMP,Ampicillin; P,Penicillin; FEP,Cefepime; CTX,Cefotaxime; CRO,Ceftriaxone; IMI,Imipenem; MRP,Meropenem; E,Erythromycin; CIP,Ciprofloxacin; LEV,Levofloxacin; CD,Clindamycin

** CLSI Interpretive criteria for susceptible*Erysipelothrix rhusiopathiae* isolates, CLSI (2016). Methods for antimicrobial dilution and disk susceptibility testing of infrequently isolated or fastidious bacteria. CLSI document M45-ED3. Clinical Laboratory Standards Institute, Wayne, PA.
