# Supplementary material for: Pathological and Genomic Findings of Erysipelothrix rhusiopathiae Isolated From a Free-Ranging Rough-Toothed Dolphin Steno bredanensis (Cetacea: Delphinidae) Stranded in Korea
Source: Front Vet Sci. 2022 May 6;9:774836. doi: 10.3389/fvets.2022.774836 (PMC9120913; doi:10.3389/fvets.2022.774836)
Supplement: Supplementary file 3 [file Data_Sheet_1.docx]

**
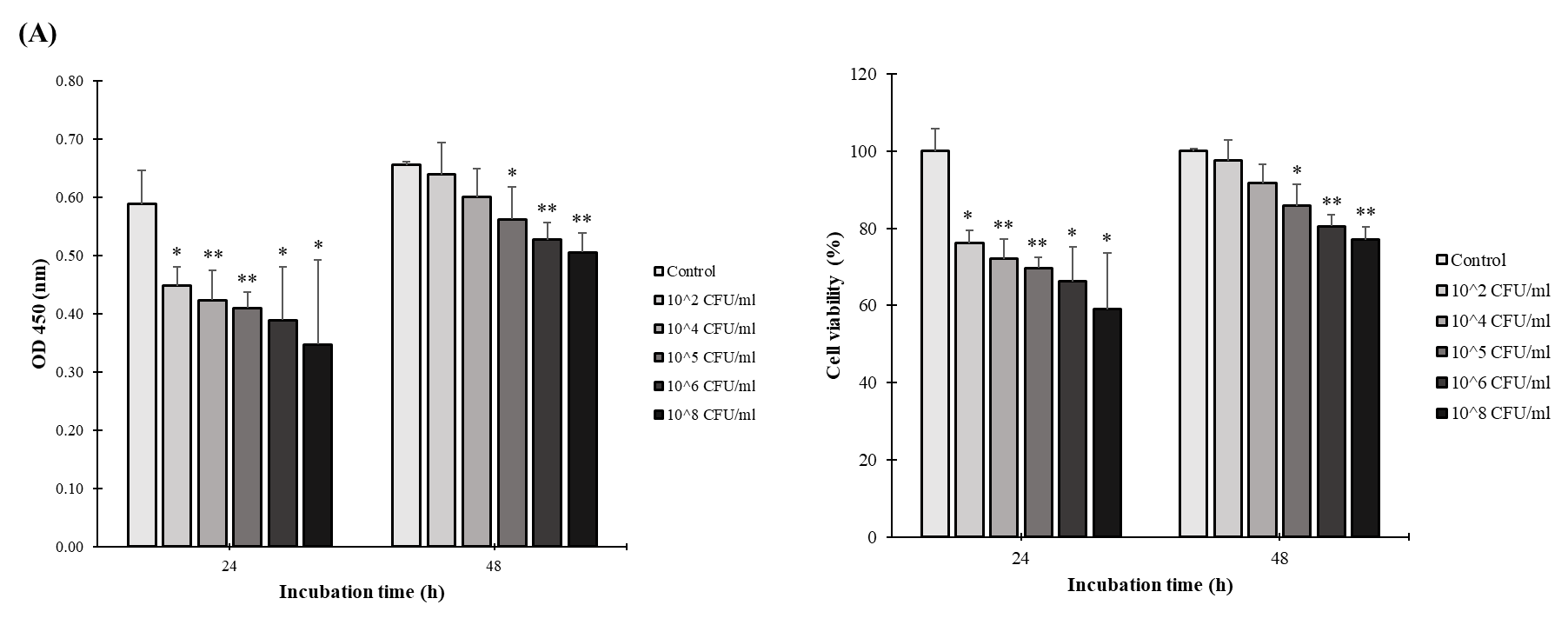
**

**
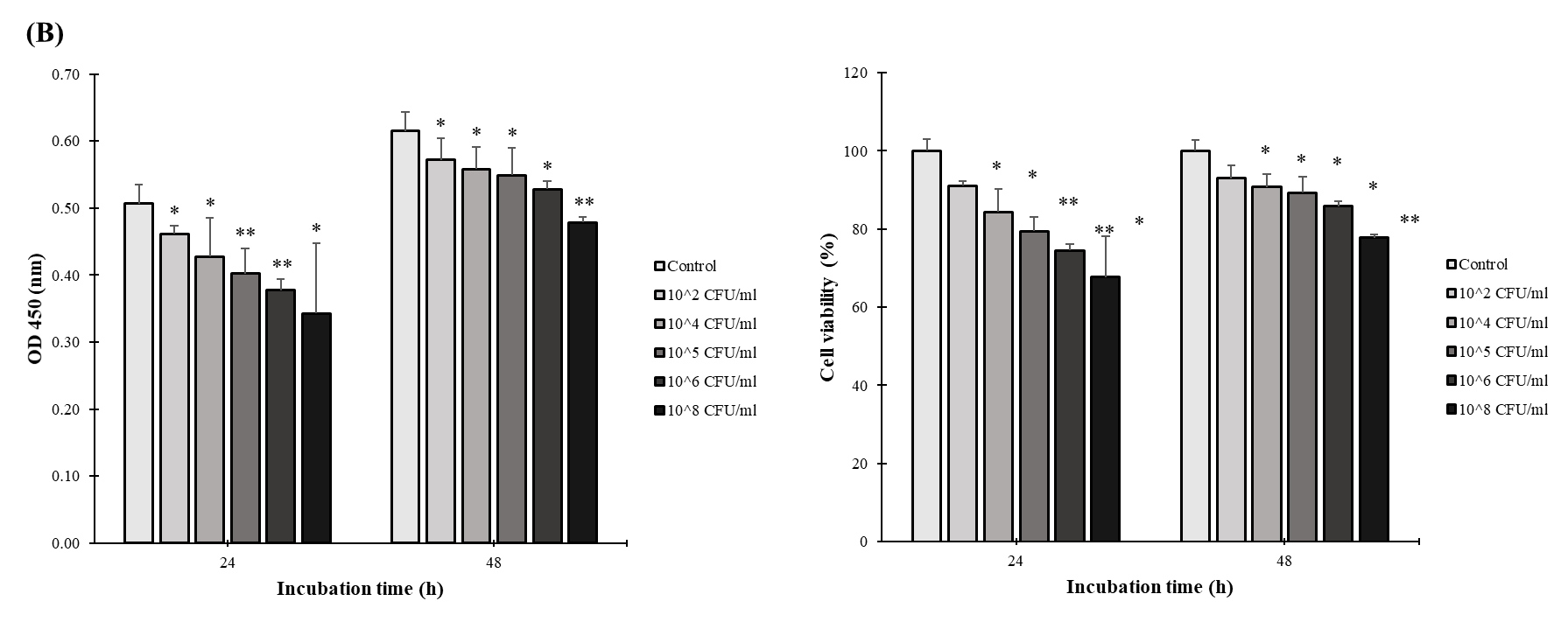
**

**Supplementary Figure S1.** (A) Raw 264.7 and (B) Calu-3 cell-viability assays were performed based on the inoculations of various concentrations (10^2^, 10^4^, 10^5^, 10^6^ and 10^8^ CFU/mL) of *Erysipelothrix rhusiopathiae* strain KC-Sb-R1 at 37°C for 24 and 48 h, respectively. Untreated cells were used as negative controls. Statistical comparisons with the control samples were performed using Student’s t-test; *p*-values <0.05 (*) and <0.001 (**) indicate statistical significance.
